# WHODAS measurement properties for women with dysmenorrhea

**DOI:** 10.1186/s12955-023-02140-y

**Published:** 2023-06-06

**Authors:** Guilherme Tavares de Arruda, Sara Giovanna de Melo Mantovan, Thuane Da Roza, Barbara Inácio da Silva, Soraia Cristina Tonon da Luz, Mariana Arias Avila

**Affiliations:** 1 Study Group on Chronic Pain (NEDoC), Physical Therapy Post-Graduate Program and Physical Therapy Department, UFSCar, São Carlos, Brazil; 2 Department of Physiotherapy, College of Health and Sport Science, Santa Catarina State University, UDESC, Florianópolis, Brazil

**Keywords:** Dysmenorrhea, Function, Disability, Women’s health, Validation study

## Abstract

**Background:**

There is an association of dysmenorrhea with human functioning and disability. However, no patient-reported outcome measure has been developed to assess this construct in women with dysmenorrhea. WHODAS 2.0 has been recognized as an important generic patient-reported outcome information of physical function and disability. Thus, the aim of this study was to assess the measurement properties of the WHODAS 2.0 in women with dysmenorrhea.

**Methods:**

This is an online and cross-sectional study conducted with Brazilian women aged 14 to 42 years with self-report of dysmenorrhea in the last three months. According to COSMIN, structural validity was evaluated by exploratory and confirmatory factor analysis; internal consistency by Cronbach’s Alpha; measurement invariance by multigroup confirmatory factor analysis between geographic regions of Brazil; and construct validity by correlating WHODAS 2.0 to the Numerical Rating Scale for pain severity.

**Results:**

One thousand three hundred and eighty-seven women (24.7 ± 6.5 years) with dysmenorrhea participated in the study. WHODAS 2.0 presented a single factor by exploratory factor analysis and adequate model by confirmatory factor analysis (CFI = 0.924, TLI = 0.900, RMSEA = 0.038), excellent internal consistence (α = 0.892) for all items and an invariancy across geographic regions (ΔCFI ≤ 0.01 and ΔRMSEA < 0.015). Correlation between WHODAS 2.0 and numerical rating scale was positive and moderate (r = 0.337).

**Conclusion:**

WHODAS 2.0 has a valid structure to assess functioning and disability related to dysmenorrhea in women.

## Background

Dysmenorrhea is the menstrual pain of uterine origin that occurs in women of reproductive age, and can be classified as primary or secondary [1]. Primary dysmenorrhea is caused by the increased of prostaglandins during menstruation, leading to muscle ischemia and uterine hypoxia. Secondary dysmenorrhea is associated with an identifiable pelvic disease such as endometriosis, adenomyosis and uterine fibroids [2].

The worldwide prevalence of dysmenorrhea is high, with 71.1% of 20,813 women reported suffering from menstrual pain [3]. In Brazil, although there are no population-based studies, existing studies report a 56-67.3% prevalence of dysmenorrhea [4, 5]. In addition, its severity can cause absenteeism/presenteeism at school/work [2, 3], increase the economic expenses with its treatment [6], and limit activities [7]. In a recent Brazilian study, the dysmenorrhea pain severity was associated with functional disability, especially general disability, mobility and participation in cases of more severe pain between 136 Brazilian women [7]. This demonstrates the importance of assessing not only the severity of dysmenorrhea, but also its interference with women’s activities. Thus, patient-reported outcome measures are needed for the valid assessment of functioning and/or disability in women with dysmenorrhea.

The International Classification of Functioning, Disability and Health (ICF), developed by the World Health Organization (WHO), describes human functioning as a term that comprises biomedical, personal, social, and environmental factors [8]. For this construct, WHO developed the World Health Organization Disability Assessment Schedule 2.0 (WHODAS 2.0), which is a generic patient-reported outcome measure that measures functioning and disability in the previous 30 days. This patient-reported outcome measure was developed based on the ICF in two different versions of 36 items (long form) and 12 items (short form). Both versions can be self-administered by the patient and had their measurement properties tested in different populations [9].

Generic instruments are usually developed to assess general population, but some particular characteristics of one specific population may go unnoticed; as such, to be adequately used, those generic patient-reported outcome measures need to be validated to one specific population and/or health condition [10]. The WHODAS 2.0 12-item version is recommended by the WHO for brief assessments of overall functioning or in populational studies which assess the factors affecting the occurrence of disability, for instance [9]. Although there is an association of dysmenorrhea with human functioning and disability [7], no patient-reported outcome measures have been developed or validated to assess this construct in women with dysmenorrhea. Therefore, this study aimed to evaluate the measurement properties of the WHODAS 2.0 in women with dysmenorrhea.

## Methods

### Design

This is a validation study that followed the recommendations of the Consensus-based Standards for the selection of health Measurement Instruments (COSMIN) [11]. The following measurement properties of the WHODAS 2.0 were assessed in women with dysmenorrhea: structural validity – degree to which the scores of an instrument are an adequate reflection of the dimensionality of the construct to be measured; internal consistency – degree of interrelation between items; measurement invariance – degree to which the performance of the items on a translated or culturally adapted instrument are an adequate reflection of the performance of the items of the original version of the instrument; and hypothesis testing for construct validity – degree to which an instrument’s scores are consistent with hypotheses based on the assumption that the instrument validly measures the construct to be measured [10]. The distribution of the WHODAS 2.0 score was also evaluated by floor and ceiling effects. This study was approved by the Institutional Ethics Committee (CAAE 52928921.2.1001.5504) and was conducted online from January to August 2022.

### Procedures and population

Participants were invited to participate in the study through Facebook®, Instagram®, WhatsApp®, emails from universities and schools through a Google Forms link. Brazilian women aged between 14 and 42 years, with a report of dysmenorrhea in the last three months and able to speak, read and write in Brazilian Portuguese were included. The lower age limit is the mean menarche of Brazilian women [5] and the upper limit decreases the probability of perimenopausal women [2]. Pregnant women, women with up to 6 months of puerperium and transgender were excluded. We excluded transgender people due to hormonal issues that may interfere with the assessment of dysmenorrhea, which was beyond the scope of this study.

The sample size calculation followed the COSMIN recommendations [10], in which 7 to 10 participants per item of the validated instrument, but greater than 100 participants is adequate. We considered the sample variability related to the Brazilian geographic census estimate for 2020 to 2021 [12], where there is 41% women living in the Southeast, 28% in the Northeast, 14% in the South, 9% in the North and 8% in the Midwest.

### Measures

#### Sample characterization questionnaire

We used a questionnaire to ask about age, region of residence (Southeast, North East, South, North, and Midwest), skin color (white, black or brown, and other), education (Elementary to high school, and College/higher education), work (yes or no), school/university (no, face-to-face only, only remotely, and mixed) and diagnosed gynecological disease (Endometriosis, Adenomyosis, Polycystic ovary syndrome, and Fibroids or uterine polyps).

#### Numerical Rating Scale

To assess the dysmenorrhea pain severity, the 11-point Numerical Rating Scale (NRS) was used. The response ranged from zero “no pain” to ten “the worst possible pain”. The numerical rating scale assessed the mean dysmenorrhea pain severity in the last period by the question “On average, how intense was the pain from your last menstrual cramp?”. In women with dysmenorrhea, the numerical rating scale had an adequate test-retest reliability (ICC = 0.90) and Smallest Detectable Change (SDC) of 2.76 points [13].

#### World health organization disability assessment schedule 2.0

The WHODAS 2.0 assesses human functioning and disability in the last 30 days. This version of the WHODAS 2.0 contains 12 items and presented excellent internal consistency (α ≥ 0.94) and test-retest reliability (ICC ≥ 0.93) for women. The answer options of the WHODAS 2.0 range from one (no difficulty) to five (extreme difficulty or cannot do it) points, with higher scores suggesting worse function [9].

### Data analysis

Studies have reported different factor structures (e.g., one-factor [14, 15], 3-factor [16], 4-factor [17], and 6-factor [18, 19]) for the 12-item WHODAS 2.0 in varied populations and health conditions that include men and women [14–19]. Thus, it was necessary to assess the structural validity of this WHODAS 2.0 version in a sample of women with dysmenorrhea by exploratory factor analysis and confirmatory factor analysis. To assess the factorability of the data, Kaiser-Meyer-Olkin (KMO) test and the Bartlett sphericity test were used. KMO ≥ 0.70 and p ≤ 0.05 in the Bartlett sphericity test indicated criterion to perform exploratory factor analysis. We performed exploratory factor analysis on the total sample, and used Minimum rank and Parallel Analysis to retain the number of factors with quartimax rotation. If necessary, items with factor loading < 0.40 were excluded [20]. For the confirmatory factor analysis, the sample was divided equally and randomly in SPSS 22. Thus, we used the maximum likelihood robust, Root Mean Square Error of Approximation (RMSEA), Standardized Root Mean Squared Residual (SRMR), Comparative Fit Index (CFI) and Tucker-Lewis Index (TLI). The model was considered adequate when RMSEA and SRMR < 0.08, CFI and TLI > 0.90 [21]. Items with higher modification indices (MI) had error covariances. Internal consistency was evaluated by Cronbach’s Alpha (α), in which α = 0.70 to 0.95 was considered adequate [22].

A multigroup confirmatory factor analysis assessed the measurement invariance between geographic regions of Brazil (Southeast, North East, South, North and Midwest) for the total sample. This analysis assessed the configural, metric, and scalar levels compared consecutively in this order. The configural invariance suggests the factorial structure to be similar across groups. For the metric invariance, factor loading of configural invariance is fixed and indicate the magnitude of the factor loadings across groups. ΔCFI ≤ 0.01 and ΔRMSEA < 0.015 were considered as signs of invariance [23].

For the hypothesis test for construct validity, Pearson correlations were calculated between the WHODAS 2.0 and numerical rating scale. We followed the correlation magnitudes by Cohen [24]: weak (r < 0.30), moderate (r = 0.30 to 0.50) and strong (r > 0.50). We hypothesized a positive and moderate correlation between human functioning/disability and dysmenorrhea pain severity [7].

The distribution of the WHODAS 2.0 score was evaluated by floor and ceiling effects. Floor or ceiling effects less than 15% were considered appropriate [25]. All analyses were conducted in Psych package in RStudio.

## Results

One thousand four hundred and thirteen people with dysmenorrhea responded to the study. Of this total, 26 (1.8%) were excluded because they were pregnant (n = 9), transgender (n = 9) and women who had given birth or had had aborted in the last 6 months (n = 8). Thus, for the evaluation of the other measurement properties of the WHODAS 2.0, 1387 women with dysmenorrhea participated in the study. For the total sample, mean age was 24.7 (± 6.5) years, most women lived in southeast Brazil (40.9%), were white (59.6%), with higher education (82.8%), worked (66%) and studies only face-to-face (48.2%). Dysmenorrhea pain severity in the previous menstrual period was 6.7 (± 2.5) points on the numerical rating scale. Table 1 shows the characteristics of the study participants for the total and split samples.

**Table 1:** Demographical and clinical characteristics of the participants

Characteristics	Mean ± SD or n (%) Total sample (n = 1387)	Mean ± SD or n (%) Split sample (n = 717)
Age (years)	24.7 ± 6.5	24.8 ± 6.4
Brazil region		
Southeast Northeast South North Midwest	567 (40.9) 346 (24.9) 248 (17.9) 113 (8.1) 113 (8.1)	291 (40.6) 183 (25.5) 123 (17.2) 67 (9.3) 53 (7.4)
Skin color		
White Black-brown Other	826 (59.6) 552 (39.8) 09 (0.6)	418 (58.3) 296 (41.3) 03 (0.4)
Education		
Elementary to high school College/higher education	239 (17.2) 1148 (82.8)	105 (14.6) 612 (85.4)
Work		
No Yes	472 (34) 915 (66)	256 (35.7) 461 (64.3)
Go to school/university		
No Face-to-face only Only remotely Mixed	161 (11.6) 669 (48.9) 160 (11.5) 397 (28.6)	79 (11) 354 (49.4) 82 (11.4) 202 (28.2)
Dysmenorrhea pain severity (NRS)	6.7 ± 2.5	6.6 ± 2.5
Gynecological diseases^a^		
Endometriosis Adenomyosis Polycystic ovary syndrome Fibroids or uterine polyps	59 (4.3) 20 (1.4) 168 (12.1) 82 (5.9)	24 (3.3) 08 (1.1) 87 (12.1) 44 (6.1)

SD: Standard deviation. NRS: Numerical Rating Scale. ^a^Participants could select more than one category

Table 1. Demographical and clinical characteristics of the participants.

In the total sample, Bartlett’s sphericity test [χ^2^(df) = 807.58(11), p < 0.0001] and KMO (0.913) were adequate. A single factor was suggested with an explained variance of 51% for WHODAS 2.0.

**Fig. 1:**
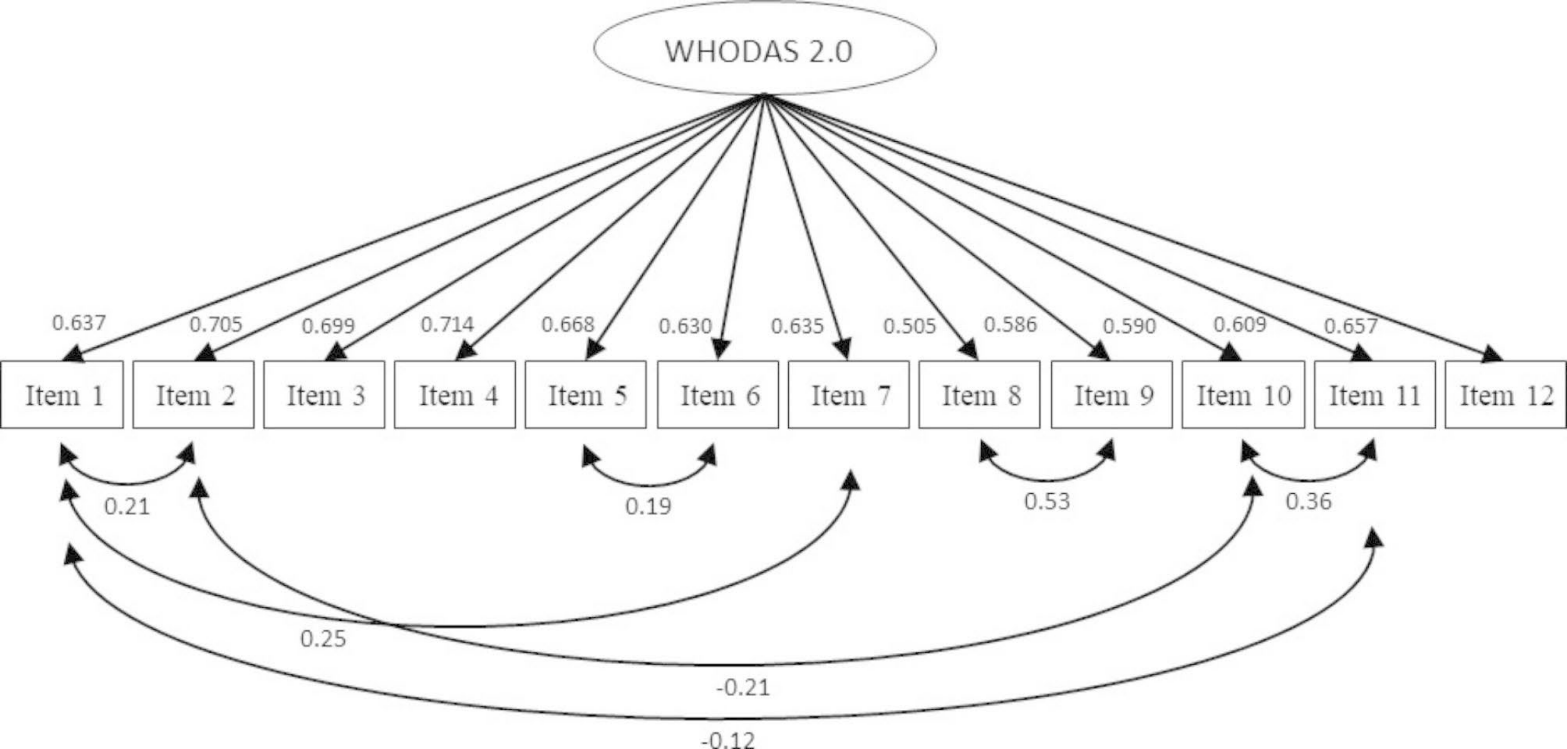
Path diagram of the WHODAS 2.0 for dysmenorrhea

Table 2 presents the factor loadings (> 0.50) and fit indexes of the WHODAS 2.0 for the split sample. The confirmatory factor analysis for showed the one-factor structure with the following fit indexes for model 1 [χ^2^(df) = 388.576(54), p < 0.001; CFI = 0.462; TLI = 0.342; RMSEA = 0.093 (90%CI 0.089–0.102); SRMR = 0.066]. To improve model fit, error covariances were freed items 8 and 9 (MI = 206.677), 10 and 11 (MI = 124.583), 1 and 7 (MI = 52.526), 1 and 2 (MI = 49.172), 2 and 11 (MI = 48.225), 5 and 6 (MI = 28.442), and 1 and 11 (MI = 22.067). This resulted in an adequately-fitting model 2 [χ^2^(df) = 93.118(46), p < 0.001; CFI = 0.924; TLI = 0.900; RMSEA = 0.038 (90%CI 0.027–0.049); SRMR = 0.029]. The path diagram of the model 2 for WHODAS 2.0 is shown in Fig. 1. Although the structure of model 2 contains correlated errors, it should be accepted as a better structure compared to model 1 due to better indexes. Internal consistence for all items was excellent (α = 0.892).

**Table 2:** Factor loadings and fit indexes of WHODAS 2.0

Items	Factor loadings Split sample (n = 717)	
	Model 1	Model 2
1. Stand up	0.655	0.637
2. Do housework	0.698	0.705
3. Learn a new task	0.695	0.699
4. Participate in community activities	0.704	0.714
5. Be emotionally affected	0.675	0.668
6. Focus for 10 min	0.637	0.630
7. Walking for long distances	0.649	0.635
8. Take a shower	0.547	0.505
9. Dress up	0.617	0.586
10. Dealing with strangers	0.602	0.590
11. Keep a friendship	0.592	0.609
12. Work	0.652	0.657
χ^2^(df) CFI TLI RMSEA (90% CI) SRMR	388.576(54) 0.462 0.342 0.093 (0.084–0.102) 0.066	93.118(46) 0.924 0.900 0.038 (0.027–0.049) 0.029

WHODAS 2.0: WHO Disability Assessment Schedule 2.0. CFI: Comparative Fit Index. df: degrees of freedom. RMSEA: Root Mean Square Error of Approximation. SRMR: Standardized Root Mean Squared Residual. TLI: Tucker-Lewis Index

Table 3 presents the Goodness-of-fit indexes for the configural, metric and scalar invariance of the multigroup confirmatory factor analysis for geographic regions of Brazil for the total sample. Differences between configural and metric model, metric and scalar model suggests the WHODAS 2.0 is invariant across geographic regions (ΔCFI ≤ 0.01 and ΔRMSEA < 0.015), which allows comparing women between these five regions.

**Table 3:** Multigroup Confirmatory Factor Analysis among geographic regions of Brazil for the WHODAS 2.0

Measurement invariance	χ^2^(df)	CFI	RMSEA	Δχ^2^(df)	ΔCFI	ΔRMSEA
**Geographic regions of Brazil (Southeast vs. Northeast vs. South vs. North vs. Midwest)**						
Configural invariance Metric invariance Scalar invariance	1337.078(270) 1417.469(314) 1507.029(358)	0.843 0.838 0.831	0.119 0.113 0.108	- 80.391(44) 89.56(44)	- 0.005 0.007	- 0.006 0.005

WHODAS 2.0: WHO Disability Assessment Schedule 2.0. CFI: Comparative Fit Index. ΔCFI: Difference in Comparative Fit Index. RMSEA: Root Mean Square Error of Approximation. ΔRMSEA: Difference in Root Mean Square Error of Approximation. df: Degrees of freedom

Correlation between WHODAS 2.0 and numerical rating scale was positive and moderate for the total sample (r = 0.337; p < 0.001). Thus, when increasing dysmenorrhea pain severity by numerical rating scale, functional disability also increases by WHODAS 2.0 total score, and vice versa. In this sample, the mean WHODAS 2.0 score was 25.4 (± 8.3) points. There were no floor and ceiling effects in the total sample (2.2% minimum and 0.1% maximum score).

## Discussion

In the current study, the structural validity, internal consistence, measurement invariance, hypothesis testing for construct validity and floor and ceiling effects of the WHODAS 2.0 were evaluated in Brazilian women with dysmenorrhea. According to exploratory factor analysis and confirmatory factor analysis, WHODAS 2.0 has a single factor and an acceptable Cronbach’s alpha. Furthermore, this patient-reported outcome measure is invariant across geographic regions of Brazil, has a moderate correlation with dysmenorrhea pain severity assessed by numerical rating scale and showed no floor and ceiling effects. This is the first study to evaluate the measurement properties of the WHODAS 2.0 for dysmenorrhea. Thus, we can only carefully compare our results with studies that included other populations [14–19, 26]. This is because dysmenorrhea specifically affects women of reproductive age, menstrual pain occurs monthly for 1 to 5 days in most cases, and pain varies greatly from woman to woman [2]. In addition, several studies that evaluated the WHODAS 2.0 measurement properties included men [14–18] and children and adolescents aged two to 16 years [19].

WHODAS 2.0 is widely used in the literature and have had their measurement properties evaluated for diverse populations and health conditions [27, 28]. In 14 studies included in a systematic review on the measurement properties of the 12-item WHODAS 2.0 among a general population and people with non-acute physical causes of disability [27], this instrument had 2 to 5 factors, high internal consistency (Alpha = 0.81 to 0.96), high correlation with other scales that assess disability, insignificant differences between repeated measures for the reliability assessment, and floor and ceiling effects were presented only in 3 studies. The widespread use of the WHODAS 2.0 generic to measure human functioning and disability may be due to its development based on the ICF, which takes into account biomedical, personal, social, and environmental factors. In addition, as it is a generic patient-reported outcome measure, it is possible to adapt it to any health condition for measuring the construct it intends to measure. Also, the short version is comprehensible to the patient and makes the assessment shorter [9]. These points make the WHODAS 2.0 a relevant patient-reported outcome measure to be used in clinical practice and scientific research.

After adjustments to the modification indices, the model tested by the confirmatory factor analysis identified a single factor in the WHODAS 2.0 structure. Although different populations and health conditions are assessed, the generic version of the WHODAS 2.0 also had a single-factor structure [9, 14, 15]. In Brazilians with Chagas disease, WHODAS 2.0 showed 3 factors with adequate global internal consistency (Alpha = 0.87) [16]. The Persian version of WHODAS 2.0 included post-injury daily life injury people and presented 4 factors with excellent total internal consistency (Alpha = 0.92) [17]. Six factors were reported in the WHODAS 2.0 versions in road traffic trauma victims in Ethiopia [18] and in children and adolescents with chronic physical illness in Canada [19]. In both studies, the internal consistency was adequate (Alpha > 0.8) [18, 19]. However, our WHODAS 2.0 version is now a valid patient-reported outcome measure to use with women with dysmenorrhea. Likewise, WHODAS 2.0 also showed an adequate internal consistency, meaning that all items are related on a single factor.

The sample size and geographic variability of the present study allowed comparing the invariance of the measure among the five geographic regions of Brazil. Our results showed that the WHODAS 2.0 has the same structure when answered by women from different geographic regions. This is a relevant point for this patient-reported outcome measure as Brazil is a huge country with cultural diversity. Thus, clinicians and researchers from each of the five geographic regions of Brazil can use the WHODAS 2.0 and find the same structure when assessing functioning and disability related to dysmenorrhea.

Regarding construct validity, our initial hypothesis was to find a positive and moderate correlation between human functioning/disability and the dysmenorrhea pain severity evaluated, respectively, by the WHODAS 2.0 and numerical rating scale. This hypothesis was accepted in this sample of women and, to the best of our knowledge, no other study has evaluated the relationship between these constructs and these patient-reported outcome measures. However, a Brazilian study using the 36-item WHODAS 2.0 reported more difficulties in the mobility and participation domains among women with more severe pain [7]. This shows that human functioning/disability is an important aspect to be evaluated in women with dysmenorrhea, but several studies only emphasize the relationship between dysmenorrhea and school absenteeism/presenteeism [3, 29].

According to COSMIN [25], the WHODAS 2.0 total score of this study did not show floor and ceiling effects. Although floor and ceiling effects do not identify the quality of a patient-reported outcome measure, these measures should be investigated. Thus, clinicians and researchers know if there are clusters of scores at a given point in the patient-reported outcome measure score. When the ceiling effect occurs, it prevents detection of improvement in a patient’s health status. On the other hand, the floor effect can impair the detection of worsening of the patient’s health condition. Thus, both effects can influence the responsiveness, which is a measurement property evaluated over time and generally assesses the change in a patient’s score to a patient-reported outcome measure after an intervention [25].

In this current study, we applied appropriate statistical methods and followed the COSMIN recommendations [11] to assess the measurement properties of the WHODAS 2.0 in women with dysmenorrhea. Furthermore, this was the first study to evaluate the measurement properties of a patient-reported outcome measure that evaluates human functioning and disability related to dysmenorrhea in a large sample of women from different geographic regions of Brazil. However, we included women with internet access due to our study design. This also reflected a high rate of women with higher education. Although we disseminated the study in schools, enrollment of women with elementary to high school was low. On the other hand, the online design allowed diversifying the sample by including women from different geographic regions of Brazil. Thus, we suggest that future studies include a larger number of women with less education. In addition, we evaluated the WHODAS 2.0 hypothesis test for construct validity with an instrument that measures another construct – dysmenorrhea pain severity. Therefore, we suggest that future studies evaluate this measurement property and report the relationship between WHODAS 2.0 and measurement instruments that measure similar constructs.

## Conclusion

The WHODAS 2.0 proved to have a valid structure and is recommended for assessment of functioning and disability related to dysmenorrhea in women. WHODAS 2.0 can simplify communication between patients and clinicians during patient assessment of the interference of dysmenorrhea-related pain on functioning and disability. Thus, this construct can have a score for the patient.

## Data Availability

Data are available at request.
